# Supplementary figures and images for: Parotid glands have a dysregulated immune response following radiation therapy
Source: PLoS One. 2024 Mar 12;19(3):e0297387. doi: 10.1371/journal.pone.0297387 (PMC10931461; doi:10.1371/journal.pone.0297387)

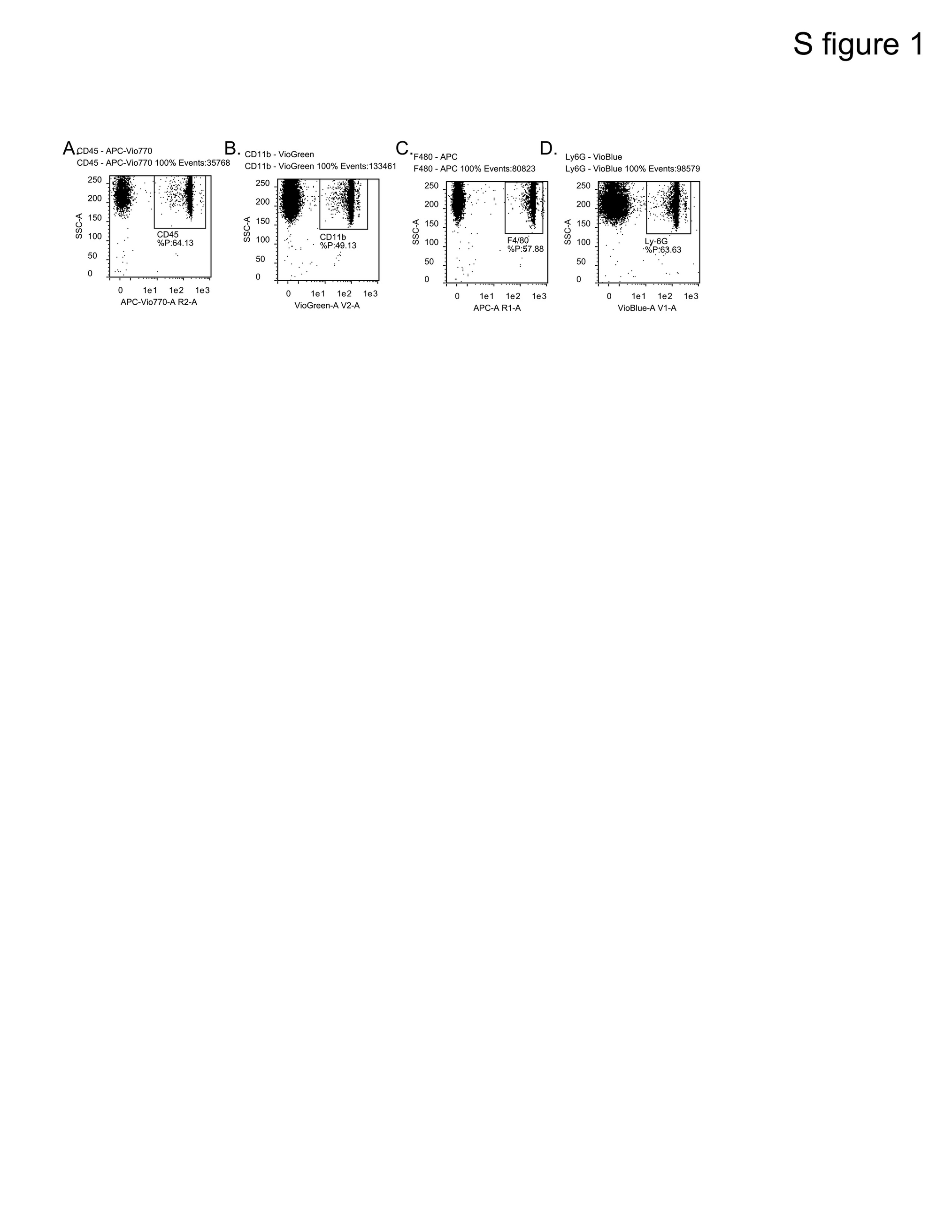

Supplement: S1 Fig — Flow cytometry data were analyzed manually via FlowLogic. Gating strategy was guided by single color controls for A) CD45, B) CD11b, C) F4/80, D) Ly-6G using anti-REA comp beads. (TIF) [file pone.0297387.s001.tif]

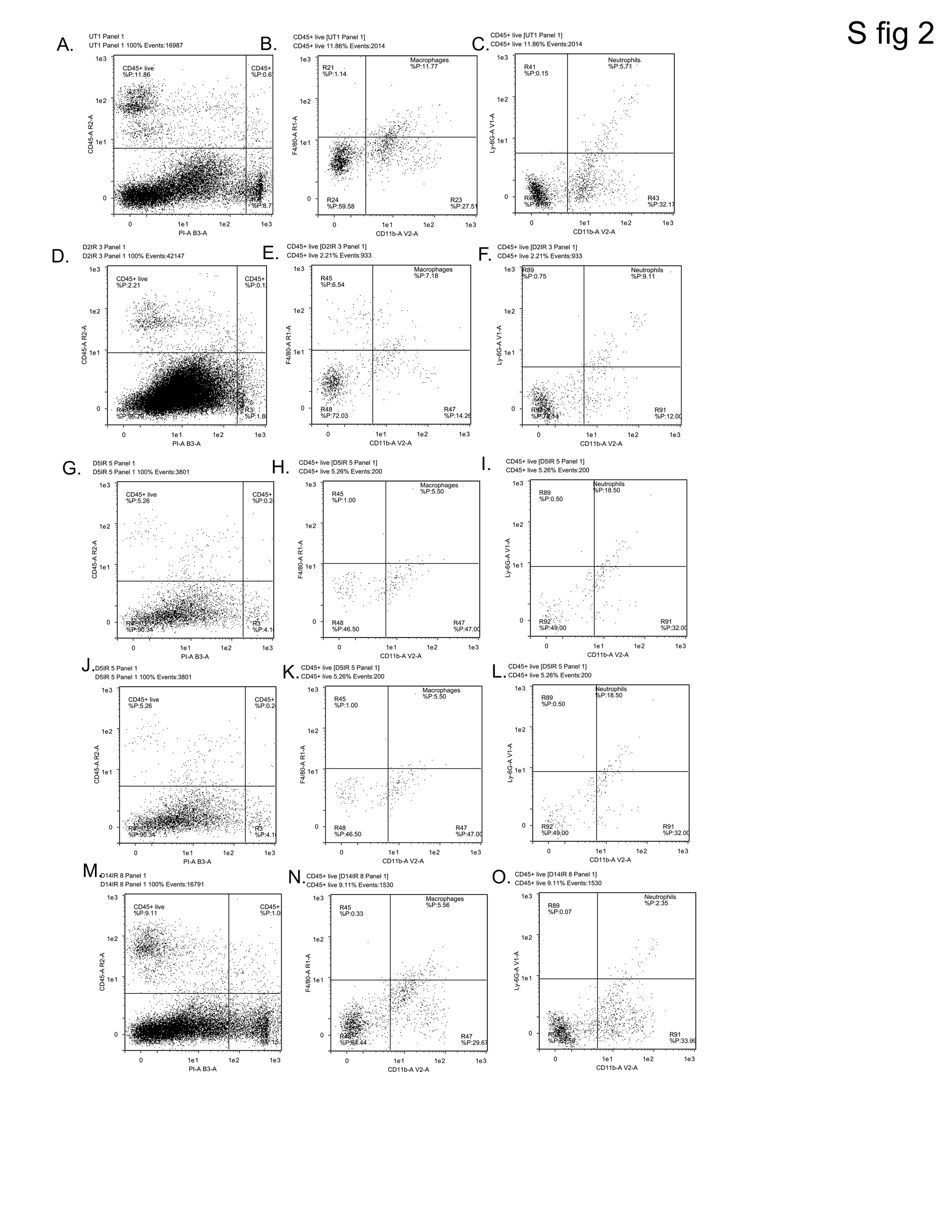

Supplement: S2 Fig — Flow cytometry data was analyzed manually via FlowLogic. Viable CD45+PI- gated from total events, F4/80+CD11b+ macrophages gated from CD45+PI-, Ly-6G+CD11b+ gated from CD45+PI- in: A-C) UT mice, D-F) D2IR mice, G-I) D5IR mice, J-L) D14IR mice, and M-O) D30IR mice. (TIF) [file pone.0297387.s002.tif]

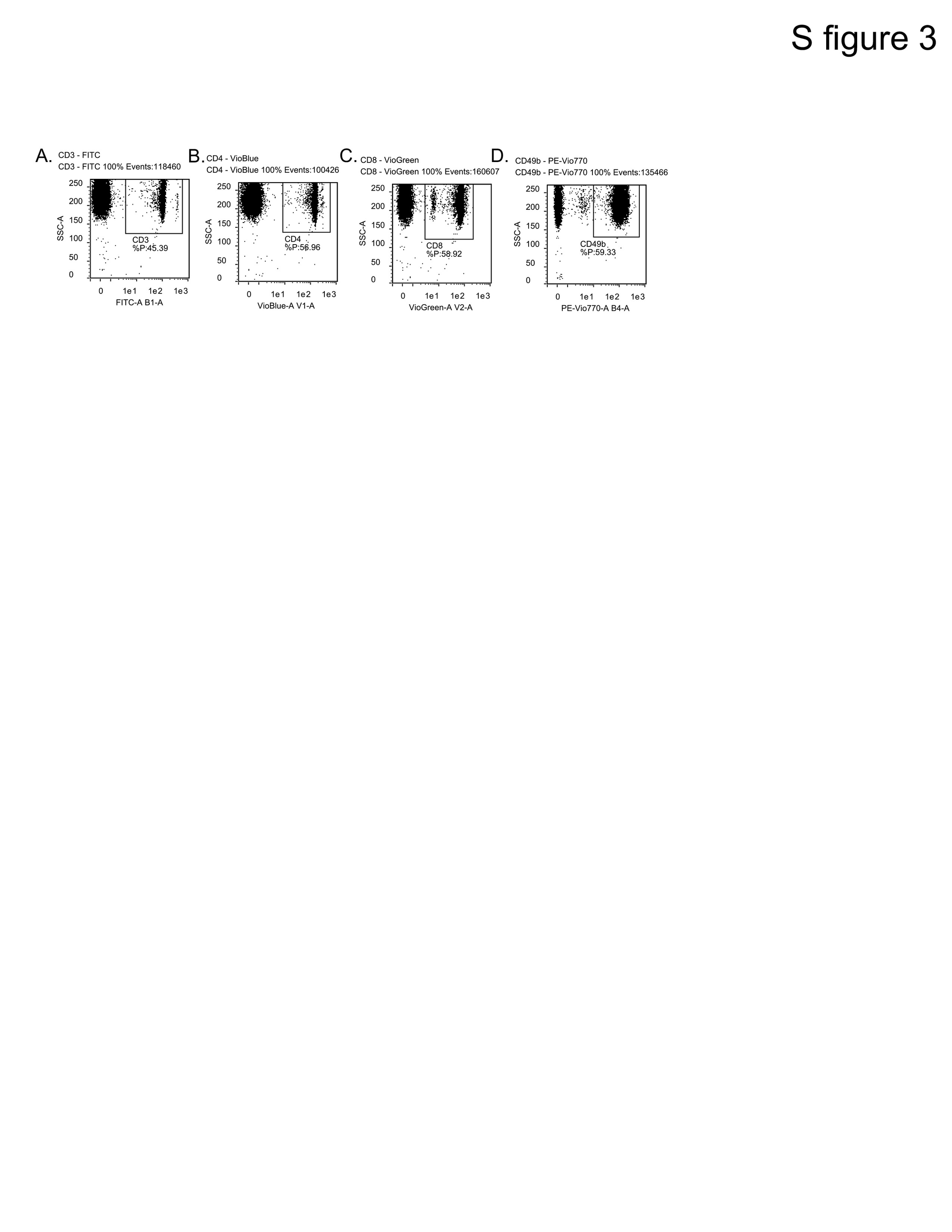

Supplement: S3 Fig — Flow cytometry data were analyzed manually via FlowLogic. Gates were determined using isotype or single color controls for A) CD3, B) CD4, C) CD8, and D) CD49b. (TIF) [file pone.0297387.s003.tif]

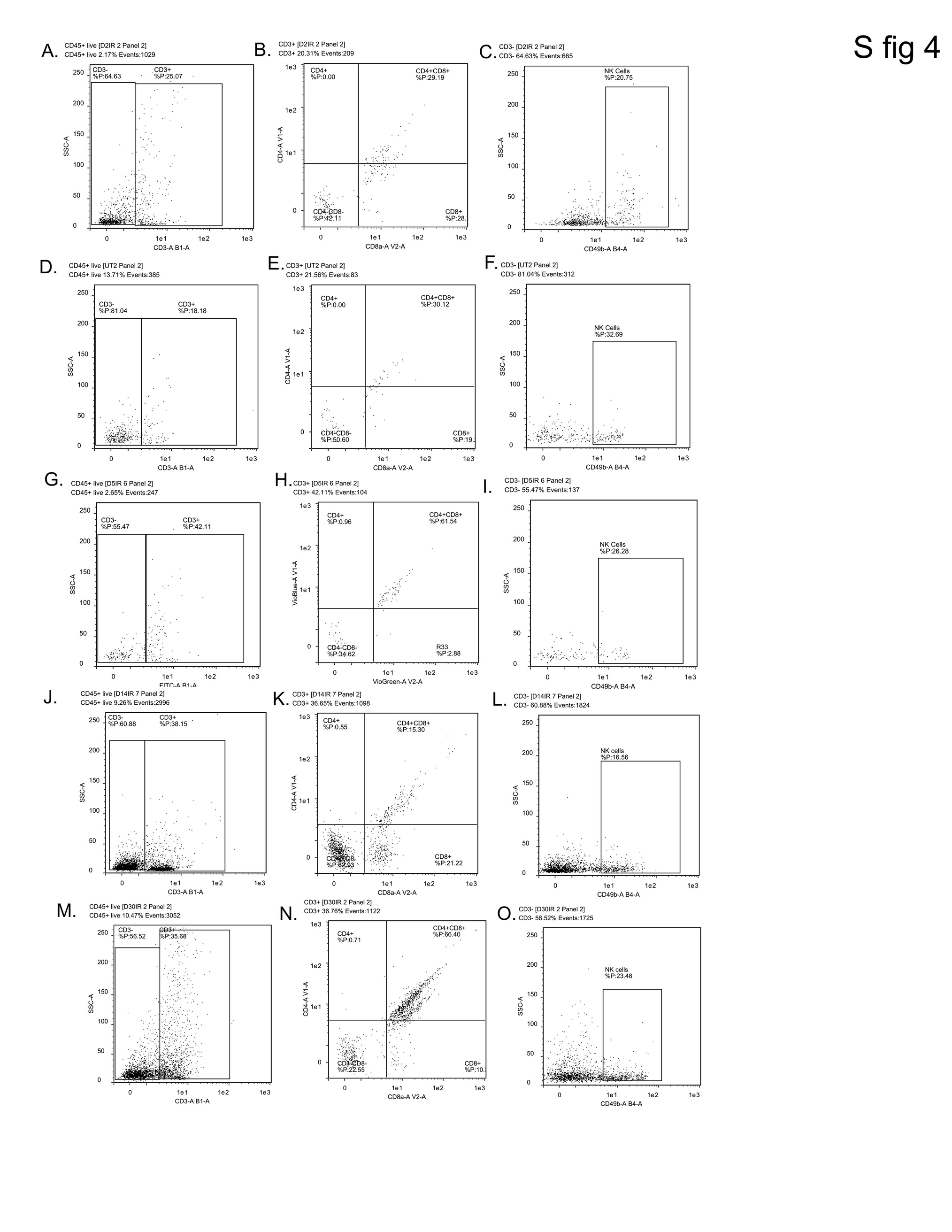

Supplement: S4 Fig — Flow cytometry data was analyzed manually via FlowLogic. CD3+ and CD3- gated from CD45+PI-, NK cells gated from CD3- cells, CD4+, CD8+, CD4-CD8-, CD4+CD8+ gated from CD3+ in: A-C) UT mice, D-F) D2IR mice, G-I) D5IR mice, J-L) D14IR mice, and M-O) D30IR mice. (TIF) [file pone.0297387.s004.tif]

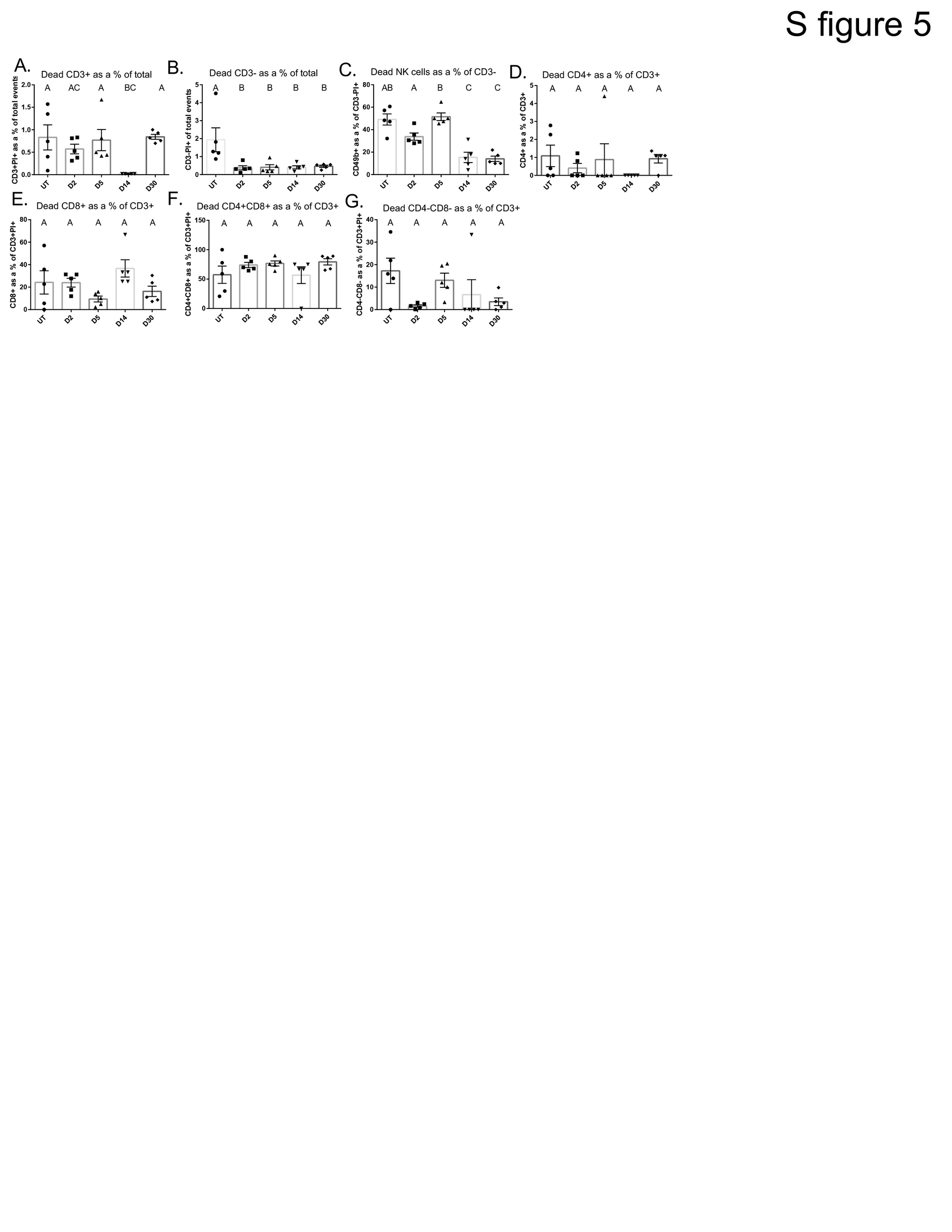

Supplement: S5 Fig — Parotid glands were prepared as described in Fig 1. A) PI+ CD3+ T cells. B) PI+ CD3- cells. C) PI+ CD49b+ NK cells. D) PI+ CD4+ T cells. E) PI+ CD8+ T cells. F) PI+ CD4+CD8+ double positive T cells. G) PI+ CD4-CD8- double negative T cells. Data were analyzed by one-way ANOVA with Tukey’s post-hoc test and represented as mean ± SEM. Treatment groups with the same letter are not statistically different from each other. (TIF) [file pone.0297387.s005.tif]

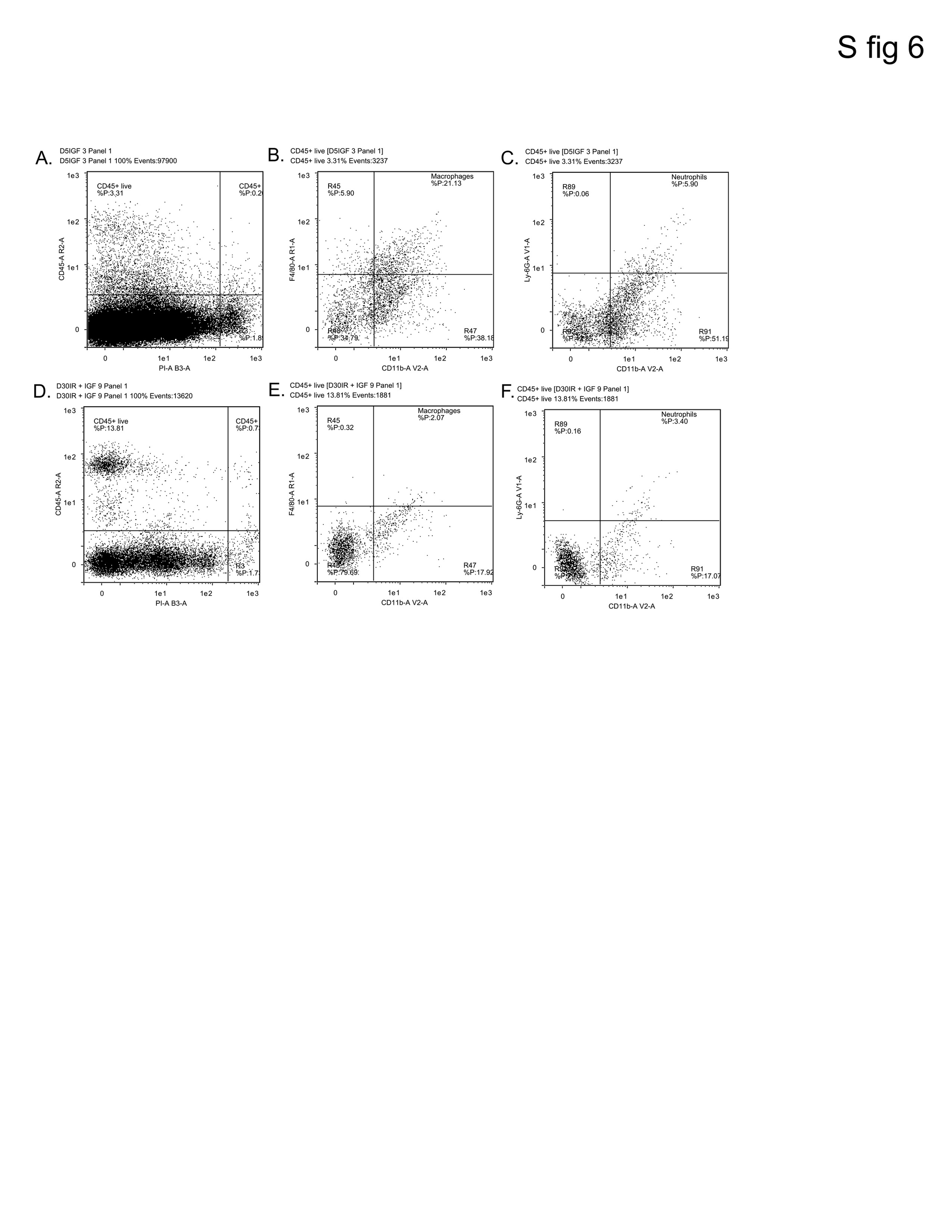

Supplement: S6 Fig — Flow cytometry data was analyzed manually via FlowLogic. Viable CD45+PI- gated from total events, F4/80+CD11b+ macrophages gated from CD45+PI-, Ly-6G+CD11b+ gated from CD45+PI- in: A-C) D5IR + IGF-1 mice, D-F) D30IR + IGF-1 mice. (TIF) [file pone.0297387.s006.tif]

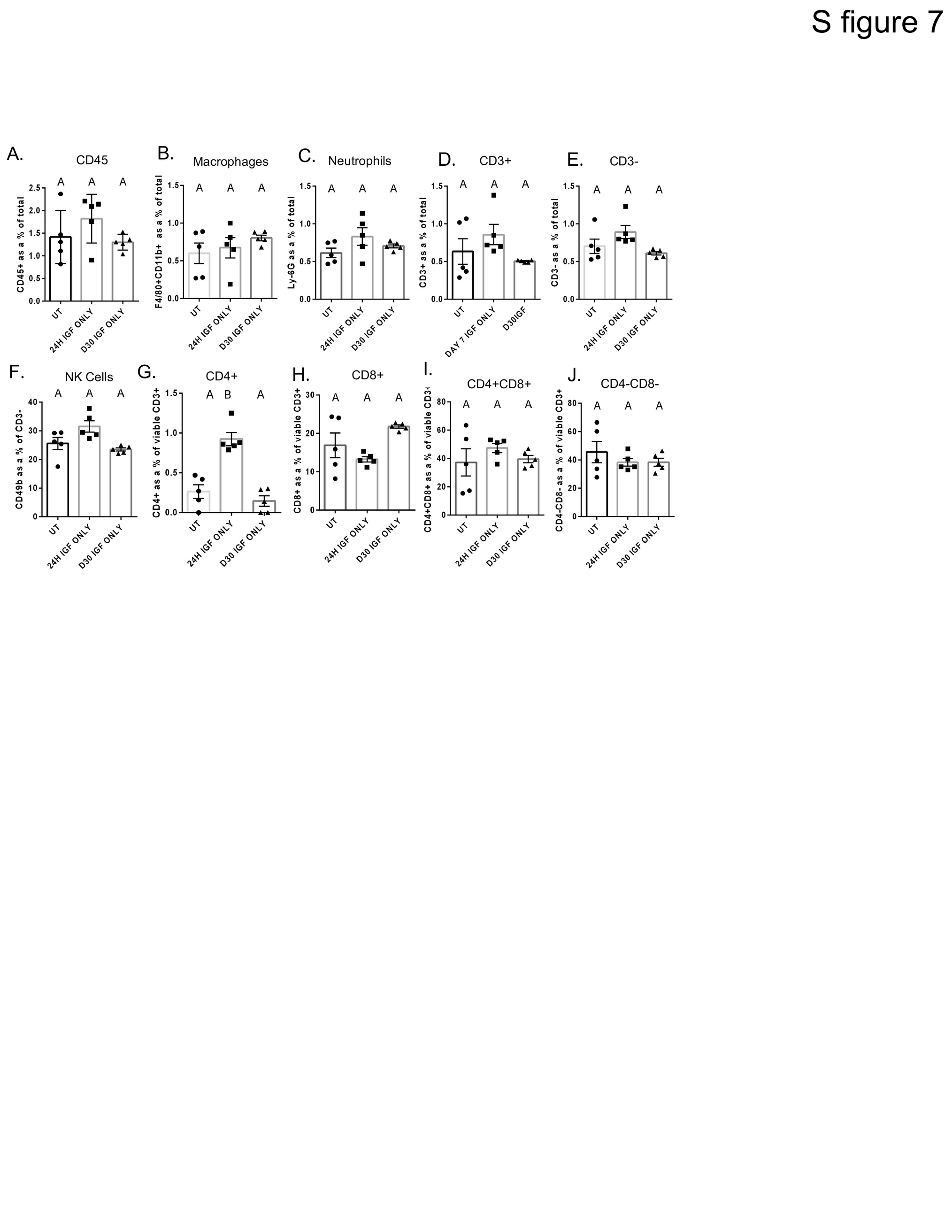

Supplement: S7 Fig — Mice were injected with IGF-1 via tail vein 3 times, 24 hours apart, and parotid tissue was dissected 24 hours after the last injection or at day 24 in order to mimic a day 30 time point. Flow cytometry data was analyzed manually via FlowLogic. Parotid tissue was prepared as a single cell suspension and labeled with antibodies for phenotyping via flow cytometry. A) Viable CD45+PI- total immune cells. B) Viable F4/80+CD11b+ macrophages, C) Viable Ly-6G+CD11b+ neutrophils. D) Viable CD3+ T cells. E) Viable CD3- cells. F) Viable CD49b+ NK cells. G) Viable CD4+ T cells. H) Viable CD8+ T cells. I) Viable CD4+CD8+ double positive T cells. J) Viable CD4-CD8- double negative T cells. (TIF) [file pone.0297387.s007.tif]

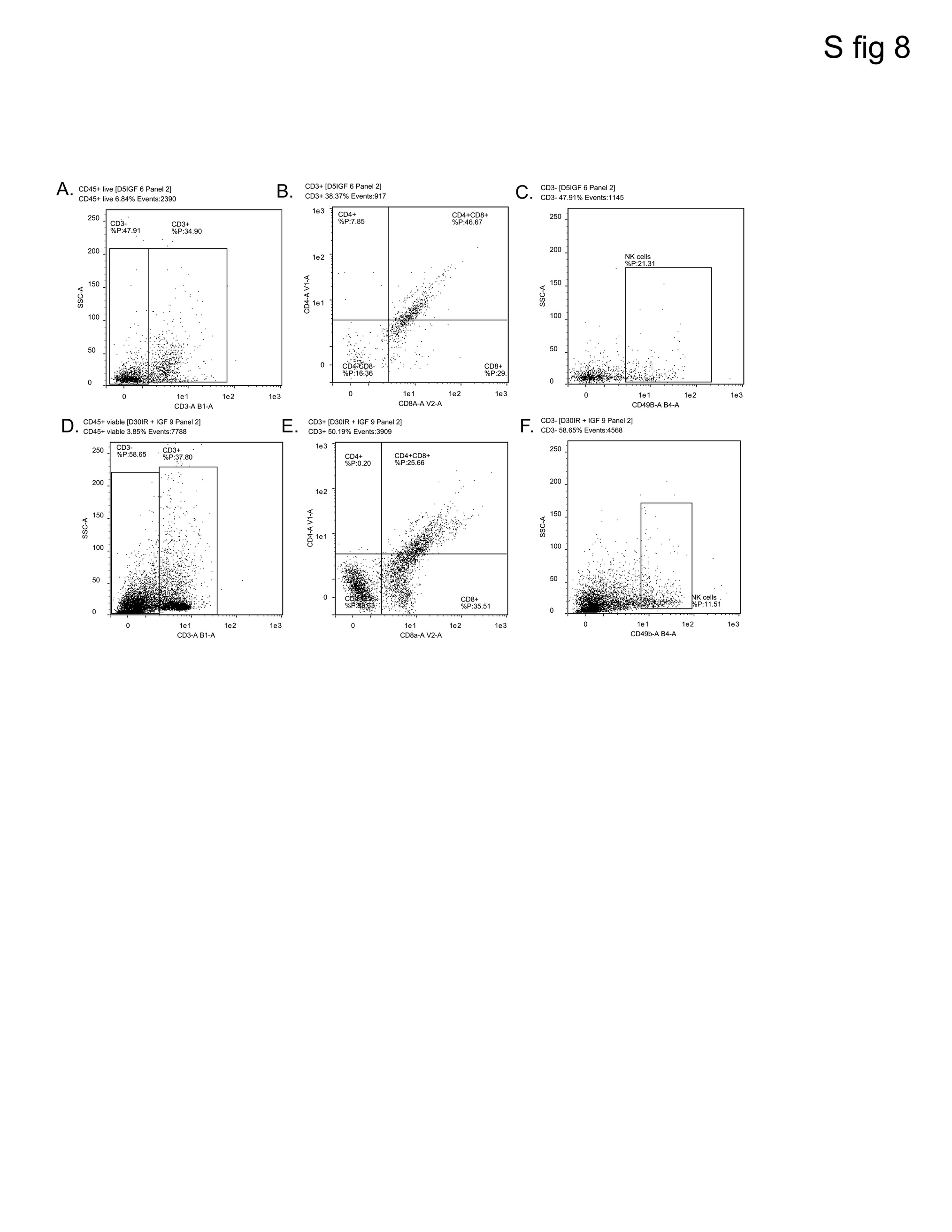

Supplement: S8 Fig — Flow cytometry data was analyzed manually via FlowLogic. CD3+ and CD3- gated from CD45+PI-, NK cells gated from CD3- cells, CD4+, CD8+, CD4-CD8-, CD4+CD8+ gated from CD3+ in: A-C) D5IR + IGF-1 mice, D-F) D30IR + IGF-1 mice. (TIF) [file pone.0297387.s008.tif]
